# Whole-Genome Sequence of Mycobacterium ulcerans CSURP7741, a French Guianan Clinical Isolate

**DOI:** 10.1128/MRA.00215-19

**Published:** 2019-07-18

**Authors:** Jamal Saad, Marine Combe, Nassim Hammoudi, Pierre Couppié, Romain Blaizot, Farah Jedir, Rodolphe Elie Gozlan, Michel Drancourt, Amar Bouam

**Affiliations:** a IHU Méditerranée Infection, Marseille, France; b Aix-Marseille-University, IRD, MEPHI, IHU Méditerranée Infection, Marseille, France; c ISEM UMR226, Université de Montpellier, CNRS, IRD, EPHE, Montpellier, France; d Equipe EA 3593, Ecosystèmes Amazoniens et Pathologie Tropicale, Université de la Guyane, Cayenne, French Guiana; e Service de Dermatologie, Cayenne Hospital, Cayenne, French Guiana; University of Arizona

## Abstract

Combined Nanopore and Illumina whole-genome sequencing of a French Guianan Mycobacterium ulcerans (Buruli ulcer agent) clinical isolate yielded a 5.12-Mbp genome with a 65.5% GC content, 5,215 protein-coding genes, and 51 predicted RNA genes. This publicly available M. ulcerans whole-genome sequence from a strain isolated in South America is closely related to M. ulcerans subsp. *liflandii*.

## ANNOUNCEMENT

Mycobacterium ulcerans is an environmental mycobacterium responsible for Buruli ulcer (1), a neglected infection currently reported in 34 tropical countries (2), including Mexico (3), Peru (4), Brazil (5), and French Guiana (6). While the whole-genome sequence of one South American isolate from French Guiana has been reported (Mu_1G897, isolated in 1988) (7), the sequence is still not available.

**FIG 1:**
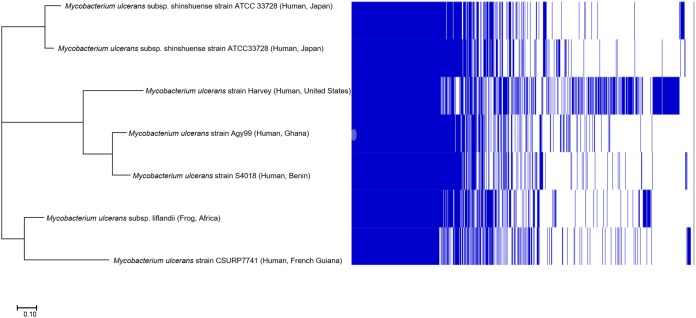
Pangenome tree incorporating Mycobacterium ulcerans strain CSURP7741 and all available complete M. ulcerans genome sequences in the NCBI database (as of January 2019).

Total DNA was extracted from several colonies of a 6-week-old subculture on Coletsos culture medium of M. ulcerans CSURP7741 using the InstaGene matrix (Bio-Rad, Marnes-la-Coquette, France) following the manufacturer’s instructions. The M. ulcerans CSURP7741 strain was initially isolated in solid Löwenstein-Jensen medium at 30°C after 8 weeks of incubation from a cutaneous biopsy specimen of the left lower limb of a 65-year-old man at the Cayenne Hospital in 2017. Total DNA (0.2 μg/μl) was sequenced using a MiSeq platform (Illumina, Inc., San Diego, CA, USA). DNA was fragmented and amplified by 12 cycles of PCR. After purification on AMPure XP beads (Beckman Coulter, Inc., Fullerton, CA, USA), the libraries were normalized and pooled for sequencing on a MiSeq instrument. Seven runs of paired-end sequencing and automated cluster generation with dual-indexed 2 × 251-bp reads were performed. The total information of 8.2 Gb was obtained from a 1,207,000/mm^2^ cluster density, and 89.3% of the cluster passed the quality control filters (10,507.2 passed filtered reads). Reads were quality checked using FastQC and trimmed using Trimmomatic version 0.36.6 (8) (SRA number ERR3335404). In parallel, MinION technology (Oxford Nanopore, Oxford, UK) was performed on one-dimensional (1D) genomic DNA sequencing using an SQK-LSK108 kit. Library AMPure XP beads (Beckman Coulter, Inc.) were constructed from 1.4 μg genomic DNA with an end-repair step and quantified using a Qubit assay (Life Technologies, Carlsbad, CA, USA). Then, 74.28 ng was loaded onto the flow cell, and 1,359 pores were activated and analyzed online using the WIMP workflow. A total of 59,875 reads were generated after a 23-minute run; 53,206 reads analyzed by the software EPI2ME yielded 130.6 Mb, an average 2.18-kb length, and a maximum read length of 68.2 kb (SRA number ERR3336325). Adding MinION reads to MiSeq reads yielded 367 contigs assembled using SPAdes software version 3.5.0 (9) with a 5,267,061-bp genome and a 65.5% GC content (ERS3388536). Contigs of under 800 bp were removed after BLASTn analysis against the NCBI database (identified as possible contaminants). Annotation using Prokka version 1.12 (10) yielded 5,266 predicted genes, 5,215 protein-coding genes, and 51 RNA genes comprising 47 tRNAs, 3 rRNA operons, and 1 transfer-messenger RNA (tmRNA). In addition, MiSeq and MinION reads were mapped with the most closely related Mycobacterium liflandii 128FXT plasmid, pMUM002 (GenBank accession number EU271968), using CLC Genomics Workbench version 7 to yield one 190,582-bp plasmid (62.6% GC content) encoding genes for mycolactone synthesis (*mlsA1* [20 kb], *mlsA2* [4 kb], and *mlsB* [16 kb]). Mapping detected IS*2404* (184 bp, 8× depth normalized with a monocopy *rpoB* gene) and IS*2606* (1,404 bp, 2× normalized depth). Genomic similarities estimated using the OrthoANI software tool version 0.93.1 (11) and *in silico* DNA-DNA hybridization estimated using the Genome-to-Genome Distance Calculator (GGDC) version 2.0 online tool (12) were, respectively, 99.49% and 94.8% with M. ulcerans subsp. *liflandii* 128FXT, 99.21% and 92.9% with M. ulcerans subsp. *shinshuense* (NZ_AP017624), 99.09% and 91.6% with M. ulcerans Agy99 (NC_008611), 99.06% and 90.9% with M. ulcerans strain Harvey (JAOL01000097), 99.08% and 91.8% with M. ulcerans strain S4018 (NZ_MDUB01000418), and 98.24% and 83% with Mycobacterium marinum E11 (NZ_HG917972). These analyses yielded an M. ulcerans pan-genome of 11,766 total genes, 3,054 conserved genes, 3,711 genes common to several species, and 5,001 species-specific genes (Fig. 1). These observations confirm clustering of South American strains with globally distributed fish isolates and M. ulcerans subsp. *liflandii*, which was responsible for an outbreak of a lethal infection in the African clawed frog, Xenopus tropicalis (7, 13). Moreover, the genetic similarities between these two isolates may orient further research on the reservoirs of M. ulcerans in French Guiana, focusing, for instance, on amphibians and, more generally, on a wide variety of freshwater species.

### Data availability.

The Mycobacterium ulcerans CSURP7741 genome sequence has been deposited at NCBI under the BioSample accession number ERS3388536. MiSeq reads have been deposited under the SRA accession number ERR3335404, and MinION reads have been deposited under the SRA accession number ERR3336325.

## References

[1] Zingue D, Bouam A, Tian RBD, Drancourt M 2017 Buruli ulcer, a prototype for ecosystem-related infection, caused by Mycobacterium ulcerans. Clin Microbiol Rev 31:e00045-17. doi:10.1128/CMR.00045-17. 29237707 PMC5740976

[2] Merritt RW, Walker ED, Small PL, Wallace JR, Johnson PD, Benbow ME, Boakye DA 2010 Ecology and transmission of Buruli ulcer disease: a systematic review. PLoS Negl Trop Dis 4:e911. doi:10.1371/journal.pntd.0000911. 21179505 PMC3001905

[3] Janssens PG, Meyers WM, Portaels F 2005 Buruli ulcer: an historical overview with updating to 2005. Bull Seances Acad R Sci Outre Mer 51:165–199.

[4] Guerra H, Palomino JC, Falconí E, Bravo F, Donaires N, Van Marck E, Portaels F 2008 *Mycobacterium ulcerans* disease, Peru. Emerg Infect Dis 14:373–377. doi:10.3201/eid1403.070904. 18325248 PMC2570831

[5] Dos Santos VM, Noronha FL, Vicentina EC, Lima CC 2007 *Mycobacterium ulcerans* infection in Brazil. Med J Aust 187:63–64. 10.5694/j.1326-5377.2007.tb01138.x 17605722

[6] Morris A, Gozlan R, Marion E, Marsollier L, Andreou D, Sanhueza D, Ruffine R, Couppie P, Guegan JF 2014 First detection of *Mycobacterium ulcerans* DNA in environmental samples from South America. PLoS Negl Trop Dis 8:e2660. doi:10.1371/journal.pntd.0002660. 24498449 PMC3907311

[7] Doig KD, Holt KE, Fyfe JAM, Lavender CJ, Eddyani M, Portaels F, Yeboah-Manu D, Pluschke G, Seemann T, Stinear TP 2012 On the origin of *Mycobacterium ulcerans*, the causative agent of Buruli ulcer. BMC Genomics 13:258. doi:10.1186/1471-2164-13-258. 22712622 PMC3434033

[8] Bolger AM, Lohse M, Usadel B 2014 Trimmomatic: a flexible trimmer for Illumina sequence data. Bioinformatics 30:2114–2120. doi:10.1093/bioinformatics/btu170. 24695404 PMC4103590

[9] Bankevich A, Nurk S, Antipov D, Gurevich AA, Dvorkin M, Kulikov AS, Lesin VM, Nikolenko SI, Pham S, Prjibelski AD, Pyshkin AV, Sirotkin AV, Vyahhi N, Tesler G, Alekseyev MA, Pevzner PA 2012 SPAdes: a new genome assembly algorithm and its applications to single-cell sequencing. J Comput Biol 19:455–477. doi:10.1089/cmb.2012.0021. 22506599 PMC3342519

[10] Torsten S 2014 Prokka: rapid prokaryotic genome annotation. Bioinformatics 30:2068–2069. doi:10.1093/bioinformatics/btu153. 24642063

[11] Lee I, Ouk Kim Y, Park SC, Chun J 2016 OrthoANI: an improved algorithm and software for calculating average nucleotide identity. Int J Syst Evol Microbiol 66:1100–1103. doi:10.1099/ijsem.0.000760. 26585518

[12] Auch AF, Jan M, Klenk HP, Göker M 2010 Digital DNA-DNA hybridization for microbial species delineation by means of genome-to-genome sequence comparison. Stand Genomic Sci 2:117. doi:10.4056/sigs.531120. 21304684 PMC3035253

[13] Tobias NJ, Doig KD, Medema MH, Chen H, Haring V, Moore R, Seemann T, Stinear TP 2013 Complete genome sequence of the frog pathogen *Mycobacterium ulcerans* ecovar *liflandii*. J Bacteriol 195:556–564. doi:10.1128/JB.02132-12. 23204453 PMC3554023

